# Multimodal Imaging in the Prenatal Diagnosis of Tuberous Sclerosis Complex

**DOI:** 10.1155/2012/925646

**Published:** 2012-10-02

**Authors:** Mariya Gusman, Sabah Servaes, Tamara Feygin, Karl Degenhardt, Monica Epelman

**Affiliations:** ^1^School of Medicine, Drexel University, Philadelphia, PA 19129, USA; ^2^Division of Radiology, The Children's Hospital of Philadelphia, 34th and Civic Center boulevard, Philadelphia, PA 19104, USA; ^3^Division of Cardiology, The Children's Hospital of Philadelphia, Philadelphia, PA 19104, USA

## Abstract

Tuberous sclerosis complex (TSC) is an autosomal dominant disorder in which benign hamartomas develop in multiple organ systems. Increasingly, stigmata of the disease, such as cardiac rhabdomyomas, are detected on routine prenatal ultrasound. Such a finding should prompt additional imaging studies in order to confirm diagnosis and to identify potential complications, which vary greatly from patient to patient. Early diagnosis allows for accurate parental counseling, coordination of high-level perinatal care, and subspecialty followup. We present a case of TSC in utero wherein access to and use of multiple imaging modalities confirmed diagnosis and allowed the patient to receive optimal care prior to birth.

## 1. Introduction

Tuberous sclerosis complex (TSC) is an autosomal dominant neurocutaneous disorder associated with benign hamartomas that can develop in almost any organ system. There is tremendous variability in the combination of organ systems affected and the degree of severity. Clinical diagnosis of TSC can be made based on the presence of major and minor features as defined by the Tuberous Sclerosis Consensus Conference [1]. The presence of two major features is sufficient for a definite diagnosis of TSC. Major features that may be discovered with prenatal imaging include cardiac rhabdomyomas, cortical tubers, subependymal nodules, and renal angiomyolipomas. The ability to detect each of these lesions differs between imaging modalities.

Suspicion of TSC in a fetus often begins with the finding of multiple intracardiac masses on routine ultrasound, suggesting rhabdomyomas. Fetal echocardiography can further delineate these masses and assess their effects on ventricular function, obstruction of blood flow, and arrhythmias. Cortical tubers and subependymal nodules are best visualized prenatally by ultrasound or MRI. Renal angiomyolipomas can be suggested by findings of fat-containing lesions with sonographic or MR imaging.

We present a case of TSC that was diagnosed prenatally with multiple imaging modalities.

## 2. Case

**Figure 1:**
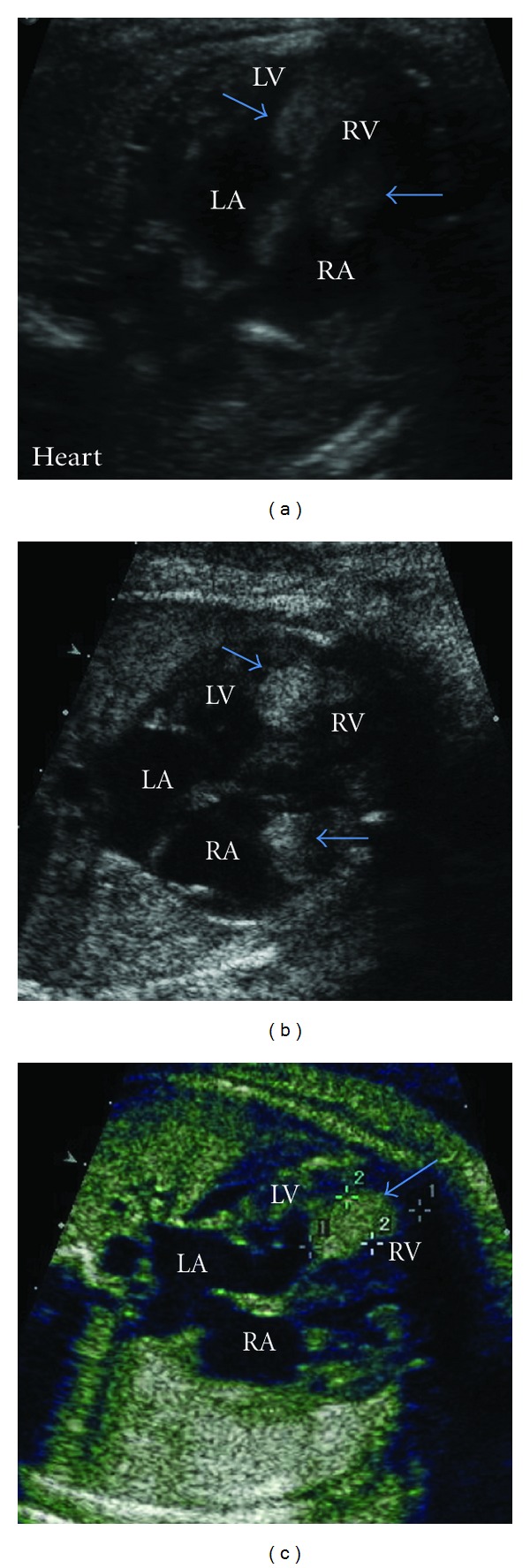
Multiple intracardiac masses (arrows) were detected on routine ultrasound (a), prompting a more thorough investigation. Fetal echocardiography ((b) and (c)) identified five masses that were within the ventricular myocardium although only two (arrows) can be seen in this plane. The masses were of uniform echogenicity and were more echogenic than the surrounding myocardium, consistent with the typical appearance of cardiac rhabdomyomas.

The mother was a 24-year-old G2P1001 with no family history of TSC or congenital defects. Fetal ultrasound at 35 weeks and 6 days demonstrated multiple intracardiac masses (Figure 1(a)), which were further visualized by fetal echocardiography (Figures 1(b) and 1(c)). The masses are seen within the ventricular myocardium and most likely represent rhabdomyomas. The masses were relatively stable in size, and ventricular function remained normal on serial echocardiography. The masses did not cause ventricular outflow or inflow obstruction. There was no evidence of fetal tachyarrhythmia.

**Figure 2:**
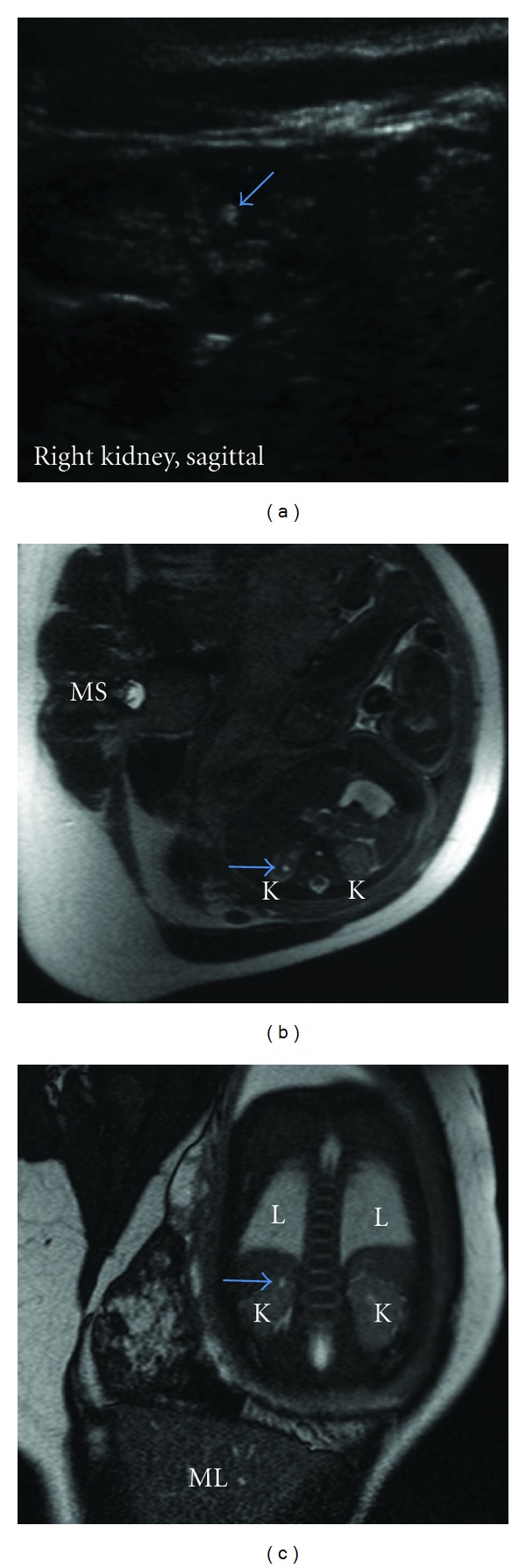
(a) On ultrasound, a single small, echogenic lesion was detected within the parenchyma of the upper pole of the right kidney, consistent with an angiomyolipoma. (b) T-2-weighted MRI image, in the transverse plane, at the level of the top of the right kidney demonstrates a high-signal lesion within the upper pole (arrow). K, kidneys; MS, maternal spine. (c) TruFISP coronal image through the kidneys shows another view of the same lesion (arrow) K, kidney; ML, maternal liver; L, lung.

Fetal ultrasound also revealed a renal lesion (Figure 2(a)). Given its increased echogenicity and solid appearance, this is most likely an angiomyolipoma. The lesion was confirmed on fetal MRI (Figures 2(b) and 2(c)).

All imaging modalities were negative for neurological findings.

The baby was born by spontaneous vaginal delivery at a children's hospital as a precaution against any cardiac complications. The possibility of arrhythmias or the development of obstruction to flow with the transition to postnatal circulation was of particular concern [2]. The perinatal course was uneventful. Postnatal echocardiography confirmed good ventricular function without inflow or outflow obstruction. Around three weeks of age, the baby developed premature ventricular contractions. Holter monitoring showed significant ventricular ectopy with an episode of nonsustained ventricular tachycardia. The ectopy responded well to the initiation of a beta-blocker. By three months of age, there was echocardiographic evidence of a decrease in the size of the ventricular masses.

In the neonatal population, TSC is most often clinically associated with seizures and dermatological findings. To date, our patient has not experienced seizures and has made normal developmental milestones, which is consistent with his lack of neurological findings on imaging. Dermatological findings are limited to a single hypopigmented macule on the lower abdomen.

## 3. Discussion

TSC may be diagnosed prenatally in the presence of two of the following: cardiac rhabdomyomas, cortical tubers, subependymal nodules, and renal angiomyolipomas.

 Cardiac rhabdomyomas are the most commonly found fetal cardiac tumor [3, 4] and are also the most common fetal finding in TSC. Larger numbers of fetal cardiac rhabdomyomas combined with a family history of TSC are more predictive of a TSC diagnosis [4]. However, only one-third of TSC cases are in children with a family history of TSC [5]. In our patient, the appearance of cardiac rhabdomyomas on ultrasound led to further imaging workup. Had the diagnosis not been made prenatally, the infant may have had more significant arrhythmias. The natural history of cardiac rhabdomyomas is for regression to occur in the first years of life. Had the diagnosis not been made prenatally, it may have been delayed by several years. 

Subependymal nodules and cortical tubers, while not the most common presenting sign, are a very frequent finding in infants receiving a TSC diagnosis (93% and 88%, resp.). Chen et al. demonstrated that ultrafast MRI can discover such lesions even when they are not apparent on a detailed sonographic examination of the fetal brain [6]. Because our patient underwent fetal MRI to evaluate his renal findings, we are confident that he did not have any subependymal or cortical masses. Consistent with this, our patient has had normal development without clinical evidence of seizures.

Renal findings, although a common manifestation of the disease, usually begin to form and increase in number during childhood and adolescence [7, 8]. There are a handful of reports of infants with severe renal disease. Only four prior articles report prenatal renal findings [9]. Our case is notable for the presence of this rare finding, which, along with the finding of multiple rhabdomyomas, confirmed the diagnosis of TSC.

Pediatricians and pediatric subspecialists should be aware of prenatal imaging findings relevant to TSC, as the presence of more than one finding is sufficient for TSC diagnosis. A suspicion of TSC based on an ultrasound finding of a cardiac, neurological, or renal mass should prompt further imaging. We utilized fetal echocardiography and MRI toward this end. Multimodal imaging allows clinicians to prepare both themselves and the family for potential complications, and arrange for early subspecialty care.
